# Neuroplasticity of Brain Networks Through Exercise: A Narrative Review About Effect of Types, Intensities, and Durations

**DOI:** 10.3390/sports13080280

**Published:** 2025-08-21

**Authors:** Carlotta Rosso, Paolo Riccardo Brustio, Jordi Manuello, Alberto Rainoldi

**Affiliations:** 1 School of Exercise and Sport Science, University of Turin, 10126 Turin, Italy; totta.rosso@gmail.com; 2 Department of Clinical and Biological Sciences, University of Turin, 10126 Turin, Italy; paoloriccardo.brustio@unito.it; 3 NeuroMuscularFunction Research Group, School of Exercise and Sport Science, University of Turin, 10126 Turin, Italy; 4 FOCUS Lab, Department of Psychology, University of Turin, 10124 Turin, Italy; jordi.manuello@unito.it; 5 Department of Medical Sciences, University of Turin, 10126 Turin, Italy

**Keywords:** neuroplasticity, brain networks, physical activity, aging, brain derived, neurotrophic factor, FMRI/SMRI, TMS

## Abstract

(1) Background: Recent decades have seen growing interest in neuroplasticity and the activity-dependent mechanisms that allow Brain Networks to adapt functionally. Among the various stimuli, physical exercise has emerged as a key modulator of brain plasticity. This narrative review aims to synthesize evidence on the structural and functional effects of physical exercise on the brain in healthy individuals aged 18–80 years. Exercise modalities were categorized into Cardiovascular, Strength, and Mixed Training. Each was further classified by intensity (Light-to-Moderate vs. Vigorous) and duration (Short- vs. Long-Term). A total of 25 interventions were analyzed to evaluate how these variables influence Brain Networks. Findings indicate that exercise type, intensity, and duration collectively modulate neuroplastic responses. Notably, physical training induces structural and functional changes in major Brain Networks, including the Default Mode Network, Salience Network, Central Executive Network, Visuospatial Network, Sensorimotor Network, and Language and Auditory Networks. These results underscore the potential of physical exercise as an effective non-pharmacological strategy to enhance brain health and plasticity across the adult lifespan. This narrative review aims to highlight the effects of physical exercise in changing the brain either functionally or structurally. Moreover, the most relevant exercise training modalities that may improve/change neural networks in healthy populations (18–80 years) were discussed. (2) Methods: Three different types of exercise were considered: (i) Cardiovascular, (ii) Strength, and (iii) Mixed Exercise. For each of them, two levels of intensity (Light-to-Moderate and Vigorous) and two durations (Short-Term and Long-Term Effects) were included. By analyzing 25 interventions, indications about the effects on the brain considering the three factors (type of exercises, intensities, and durations) were provided. (3) Results: The findings suggest that the type of exercises, intensities, and durations could to lead neural modification over time. Specifically, exercise intervention contributes to both structural and functional changes in brain regions located in key Brain Networks, including the Default Mode Network, Salience Network, Central Executive Network, Visuospatial Network, Sensorimotor Network, and Language and Auditory Networks. (4) Conclusions: In conclusion, the evidence presented herein underscores the beneficial effects of physical exercise on the structural and functional integrity of the brain, highlighting its importance as a non-pharmacological intervention to improve brain plasticity.

## 1. Introduction

The human brain, observed on a micro-scale level, is an organ consisting of about 9 × 10^10^ specialized cells, called neurons, which are able to receive and transmit electrical signals on a scale of a few milliseconds [1]. At the macro-scale level, several regions can be identified that are constantly processing and sharing information with each other. These complex relationships define what are commonly called neural networks [1].

One of the most peculiar and relevant properties of the brain, both on the micro and macro scales, is its ability to respond to intrinsic or extrinsic stimuli by reorganizing its structure, function, and connections [1]. Physical activity represents a promising non-pharmacological interventional approach to maintaining, delaying, and/or improving brain structure and function [2,3,4,5]. This phenomenon, which persists throughout the lifespan, is termed neuroplasticity [6], and involves the entire Central Nervous System (CNS). Notably, it offers a convenient framework for understanding both normal and pathological psychological function [7]. In fact, while neuroplasticity plays a fundamental and beneficial role in shaping the brain [6], maladaptive plasticity could explain many developmental, acquired, and neurodegenerative brain disorders [7]. Furthermore, the functional and structural architecture of Brain Networks, such as the Default Mode, Salience, and Sensorimotor Networks, are related to behavioral and psychological outcomes. This includes cognitive adaptability and motor expertise [8], as well as mental health and quality of life measures associated with regular exercise habits [9]. These findings support the relevance of studying Brain Networks as a key dimension of the neuroplastic changes induced by physical exercise.

In the last few decades, there has been great interest in activity-dependent plasticity, which sets neural circuits into new functional conditions [7]. Among different mechanisms to induce such modifications, physical exercise, a specific form of physical activity, is broadly defined as a structured routine exercise designed to improve or maintain physical fitness [10], where exercise type, intensity, and duration must be detailed. Additionally, the goal of learning a new motor task leads to a reorganization of neural circuits, and the ability to perform the learned movement persists even in the absence of continuous training, suggesting that motor skills are encoded as lasting neurobiological changes within the CNS [11]. The literature highlights that morphological and functional modifications can be found even during aging [12,13,14], showing that exercise activity is able to attenuate age-related impairments, not only on the physical side but also on the cognitive side [2,3,12,13,14]

Although most of the current psychological and neuroscientific research describes findings at the level of Brain Networks, this is less common in exercise-related domains, i.e., when neural changes are correlated and due to physical exercise type, intensity, and duration. Studies suggest that exercise can induce structural changes in different brain regions and modulate functional connectivity between brain areas, thereby improving brain function [2,3,4,5,14,15].

Based on these findings, the aim of this narrative review is to synthesize the evidence on the structural and functional effects of physical exercise on brain regions, and to organize the findings so as to elucidate their meaning on a Brain Network level in healthy individuals aged 18–80 years, categorizing exercise modalities by type, intensity, and duration. Given the variability in study designs, participant populations, exercise interventions, and neuroimaging methods, a systematic or quantitative synthesis was not feasible for the scope of this work. We therefore adopted a narrative review approach to provide a broad and conceptually integrative overview of a highly heterogeneous body of literature. To structure our synthesis, we classified the selected studies according to three main exercise modalities (i.e., Cardiovascular, Strength, and Mixed), across two intensity levels (Light-to-Moderate and Vigorous) and two durations (Short-Term and Long-Term Effects). This narrative approach allows us to explore and summarize potential improvements/changes in Brain Networks and how different exercise types, intensities, and durations influence structural and functional changes among brain regions. Based on the concurrent literature, we hypothesize that different exercise types, intensities, and durations can be considered mediators of brain health by positively affecting both the structural and functional dimensions of the brain.

## 2. Materials and Methods

### 2.1. Methodology

Relevant articles were selected based on their focus on adult populations and the use of imaging or electrophysiological techniques to assess neuroplasticity. The following inclusion criteria were adopted: (1) participants were healthy adults with an overall age range between 18 and 80 years; (2) absence of any mental, psychological, physical, or motor disorders; (3) network activity and neuroplasticity measured objectively using functional or Structural Magnetic Resonance Imaging (FMRI/SMRI) or electromyography (EMG) in conjunction with transcranial magnetic stimulation (TMS); and (4) articles written only in the English language. Studies that relied solely on EMG or investigating only acute effects were excluded. Other sources (e.g., reviews, meta-analyses, abstracts, opinion articles, books, statements, letters, editorials, comments, and non-peer-reviewed journal articles) were excluded.

A comprehensive literature search was conducted on major biomedical databases (i.e., PubMed and MEDLINE). The selection process was conducted by one author (C.R.), who independently screened the title and/or the abstract of the selected studies to identify studies that potentially met the inclusion criteria. The full texts of potentially eligible studies were then assessed for eligibility by the same author (C.R.). Articles were initially selected, and in cases where there was uncertainty about their inclusion, a second author (P.R.B.) was consulted for the final decision. The research included articles published from 2004 to November 2023. The last search update was conducted on 31 May 2025. Thus, a narrative synthesis approach was employed to summarize and analyze the findings from the included studies. The total number of articles reviewed was 65 after applying the inclusion and exclusion criteria.

### 2.2. Criteria for Conceptual Classification

To synthesize the findings meaningfully, the included studies were classified according to three key dimensions, (1) exercise type (Cardiovascular, Strength, or Mixed), (2) intensity (Light-to-Moderate or Vigorous), and (3) duration (Short- vs. Long-Term). This classification framework, aligned with ACSM guidelines [16], provides a structured interpretation of the potential impact of different exercise modalities on neuroplasticity-related outcomes. Of note, to avoid the excessive fragmentation of studies in terms of intensity, we decided to consider Light and Moderate Intensities together.

Cardiovascular Exercise was defined as regular, purposeful exercise, performed in one (continuous) session per day or in multiple sessions of ≥10 min, which involves major muscle groups and is measured using the HR_max_ or VO_2max_ [17]. This type of exercise involves different training methodologies including walking activity or running [18,19], as well as high-intensity interval training [17,20].

Strength Exercise was defined as resistance-based training (performed with a variety of types of exercise equipment and/or body weights), involving each major muscle group and measured by counting maximal repetitions and, consequently, the work percentage required [16]. Strength-specific programs incorporate the use of concentric, eccentric, and isometric muscle actions, either individually or in combination, as well as the performance of bilateral and unilateral single- and multi-joint exercises [21].

Mixed Exercise was characterized as a form of exercise activity integrating motor skills (e.g., balance, agility, coordination, and gait), proprioceptive exercise training, and multifaceted activities (e.g., Tai Chi), all aimed at contributing to overall physical fitness and well-being [22].

Considering exercise intensity, Light-to-Moderate Intensity was defined as ranging between 57% and 76% of the Maximum Heart Rate (HR_max_) or 37 and 64% of the Maximal Oxygen Consumption (VO_2max_ ) for cardiovascular activity, and between 30% and 70% of 1 RM for strength training. For Vigorous Intensity, cardiovascular parameters ranged from 76% to less than 96% of the HRmax or from 64% to less than 91% of the VO_2max_, while strength training fell between 70% and less than 85% of 1 RM [16].

Finally, the duration of exercise interventions was operationally classified into Short-Term and Long-Term. Studies lasting less than one year were considered Short-Term interventions, while those with a duration of one year or longer were classified as Long-Term.

## 3. Results

### 3.1. Overview of Evidence

#### 3.1.1. Study Description: Types of Physical Exercise and Brain Networks

In terms of population, fourteen (64%) interventions included young adults (<35 years) and eleven (36%) included older people (≥60 years). In general, about half of the subjects included in the studies were female. Two out of the thirteen studies were based on Cardiovascular Exercise, and one was based on Strength Exercise, focusing on Long-Term effects and including older athletes. Similarly, two other Mixed Exercise articles on young people focused on Long-Term effects and included artistic gymnasts. In the Short-Term training protocol, the average frequency was two to five times a week, except for two papers with a duration ranging from 15 to 90 min [18,23]. In the Long-Term training, the training protocol’s average frequency, when reported, was two to three times a week. The duration range was reported only in one paper [24].

The intensity of the Cardiovascular Exercise, which included activities such as walking, running, swimming, and cycling, was Light-to-Moderate in six papers (five with a Short-Term training protocol and one with a Long-Term one); Vigorous in five articles (each one a with a Short-Term training protocol); and not reported in two papers (one with a Short-Term and the other with a Long-Term training protocol). The intensity of the Cardiovascular Exercise was measured either via HR (ranging from 40 to 85% of the HRmax) or via oxygen uptake (~80% of the VO_2max_ ), with exercise durations ranging from 15 to 60 min per session. As for Strength Training, the intensity was consistently Vigorous Intensity across studies—two employing Short-Term training protocols and one using a Long-Term protocol—with reported intensities ranging from 80 to 85% of 1 RM (or 80% of the maximal voluntary contraction [MVC] for isometric exercises). The exercise duration was reported in only one study, ranging from 15 to 20 min [25].

In Mixed Exercise interventions, which included activities such as artistic gymnastics [26,27], dancing [22,28,29], Tai Chi [22], cycling associated with strength endurance and flexibility [29], Nordic walking and stretching and flexibility exercises [30], juggling [31] and Karate [32], the intensity was measured based on the specific physical activity performed. Exercise durations ranged from 15 to 90 min. In two studies, the intensity was classified as Light-to-Moderate (both employing Short-Term training protocols), and in two studies it was classified as Vigorous (also with Short-Term protocols).

The following paragraphs describe the findings obtained for brain regions located in seven Brain Networks, for White Matter and Subcortical Gray Matter, and for the Corticospinal Tract. The brain changes described in these studies were both structural and functional. The structural brain changes consisted overall of an increase in volume, tissue density, and vascular plasticity in localized brain regions, whereas functional changes were reported for connectivity, excitability, and activation.

#### 3.1.2. Papers’ Subdivisions for Each of the Seven Brain Networks, White and Subcortical Gray Matter, and Corticospinal Tract

##### Default Mode Network, Salience Network, and Central Executive Network

The Default Mode Network (DMN) consists of bilateral, symmetrical cortical areas in the medial and lateral parietal, medial prefrontal, and medial and lateral temporal cortices of the human brain [33]. Nineteen papers [17,19,20,22,23,24,34,35,36,37,38,39] consisting of twenty-one interventions investigated the effect of exercise on the DMN. Out of the twenty-one interventions, twelve were based on Cardiovascular Exercise (~57%). Half used a Light-to-Moderate Intensity, five a Vigorous Intensity, and one did not report the intensity. The largest portion of them (*n* = 11) were focused on Short-Term effects, whereas only one focused on Long-Term effects. The HR (40–85% HR_max_) was the most used parameter to evaluate exercise intensity, followed by two studies that used repetitions per minute (RPM) (60–70 RPM corresponding to 120–170 beats per minute (BPM)) and one that used the VO_2max_ (80%VO_2max_). One did not report the exercise intensity measurement methods. One intervention focused on Strength Exercise and proposed a Vigorous Intensity (70–85% 1 RM) for a period of 52 weeks (Long-Term effect). Finally, eight interventions (38.0%) focused on Mixed Exercise. Of these, one quarter reported Light-to-Moderate Intensity (measured via HR with a range of 50–69% HR_max_), another quarter Vigorous Intensity (measured via the Physical Working Capacity (PWC) with an estimated HR_max_ range of 82–86%), while the remaining half did not report any intensity. Of these interventions, the majority (*n* = 7) evaluated Short-Term effects, while only one intervention evaluated Long-Term effects.

Of the twenty-one interventions analyzing the Default Mode Network (DMN), six studies reported functional changes, eleven structural changes, and four reported both.

Functional changes were primarily observed in studies such as those of Colcombe et al. [39], Voss et al. [24], and Tomita et al. [27], where resting-state FMRI and task-based FMRI revealed increased functional connectivity and activation in core DMN regions, especially the hippocampus and posterior cingulate cortex, following Cardiovascular or Mixed Exercise programs. For example, Colcombe et al. [39] reported that six months of Cardiovascular Training led to greater DMN engagement during dual-task conditions in older adults, while Voss et al. [24] documented improved within-network coherence in medial temporal structures. In contrast, Motes et al. [34] described a reduction in PFC activation during working memory tasks, interpreted as increased neural efficiency.

Structural changes were predominantly assessed using voxel-based morphometry (VBM) and diffusion tensor imaging (DTI). Studies such as Erickson et al. [14,37], Rehfeld et al. [29], and Lehmann et al. [17,20] found increased Gray Matter volume in the hippocampus, greater cortical thickness in the medial PFC, and an enhanced White Matter microstructure in DMN-related pathways. Additionally, Maass et al. [19] reported greater cerebral blood flow in older adults after a Vigorous-Intensity exercise intervention, suggesting enhanced vascular plasticity.

The neuroimaging techniques used across these studies included resting-state and task-based FMRI for functional changes, and SMRI, VBM, DTI, and arterial spin labeling for detecting structural and perfusion-related modifications.

The Salience Network (SN) is a large-scale Brain Network involved in orienting attention to internal and external stimuli [40,41]. According to Smitha et al. [], its cortical regions are the insula and the cingulate cortex [33]. In this network, twelve studies [17,20,22,27,29,30,31,32,34,38,39] consisting of fourteen interventions showed structural or functional changes depending on the analyzed studies. Out of the fourteen interventions, six were based on Cardiovascular Exercise (~48%). Half used a Light-to-Moderate Intensity, two a Vigorous Intensity, and one did not report the intensity. All focused on Short-Term effects. The HR (ranging from 40 to 75% HR_max_) was the most used parameter for evaluating exercise intensity, followed by the RPM (*n* = 2, 60–70 RPM corresponding to 120–170 BPM). One did not report the exercise intensity measurement methods. One intervention focused on Strength Exercise and proposed a Vigorous Intensity (70–85% 1 RM) for a period of 52 weeks (Long-Term effect). Finally, half of the fourteen interventions focused on Mixed Exercise. Out of these, two reported Light-to-Moderate Intensity (measured via HR with a range of 50–69% HR_max_), another two Vigorous Intensity (PWC with an estimated HR_max_ range of 82–86%), while three did not report any intensity. Most of these interventions (*n* = 6) evaluated Short-Term effects, while only one intervention examined Long-Term effects.

The involvement of the Central Executive Network (CEN) or Executive Control Network (ECN), responsible for high-level cognitive functions such as planning, decision making, executive functions and control, and the control of attention and working memory [42], was described in eleven studies [17,18,20,22,27,29,30,31,32,34,39] consisting of thirteen interventions. Out of the thirteen interventions, six were based on Cardiovascular Exercise (~46%). Half used a Light-to-Moderate Intensity, two a Vigorous Intensity, and one did not report the intensity. Each of them focused on Short-Term effects. In half of the interventions, intensity was measured via HR (ranging from 40 to 75% HR_max_), two via RPM (60–70 RPM corresponding to 120–170 BPM), and one did not report the exercise intensity measurement methods. No intervention focused on Strength Exercise. Finally, seven interventions (~54%) involved Mixed Exercise. Within this group, two reported Light-to-Moderate Intensity (measured via HR with a range of 50–69% HR_max_), two Vigorous Intensity (measured via PWC with an estimated HR_max_ range of 82–86%), while three did not report any intensity. Of these interventions, the majority (*n* = 6) evaluated Short-Term effects, while only one intervention evaluated Long-Term effects.

Of the fourteen interventions analyzed for the SN, four studies reported only functional changes, primarily consisting of increased activation and functional connectivity within Salience-related brain regions such as the anterior insula and anterior cingulate cortex, as measured via task-based and resting-state FMRI. Some studies also noted a reduction in PFC activation during cognitive tasks, requiring inhibitory control. Seven interventions reported only structural changes, assessed through VBM and diffusion tensor imaging DTI. These included increased Gray Matter volume in Salience-related areas, particularly the parietal and cingulate regions, and enhanced White Matter integrity and microstructure changes. Additionally, some studies indicated improvements in vascular plasticity, inferred from increased cerebral perfusion. Three interventions analyze both functional and structural modifications. The neuroimaging methods employed included FMRI for functional data and SMRI, VBM, and DTI for structural data.

##### Visuospatial Network and Sensorimotor Network

The Visuospatial Network (VSN), anchored in intraparietal sulci and frontal eye fields, is formed via synchronous activations at the posterior parietal cortex of the pita-parietal junction, midline of the precuneus, and posterior cingulate cortex, and the frontal pole [33,43,44] was studied in ten papers [18,20,22,26,27,29,30,31,32,39] consisting of twelve interventions. Out of the twelve interventions, four were based on Cardiovascular Exercise (~33%). Half used a Light-to-Moderate Intensity, one quarter Vigorous Intensity, and another quarter did not report the intensity. In terms of measurement, half were assessed using HR (ranging from 40 to 70% HR_max_), a quarter RPM (60–70 RPM corresponding to 120–170 BPM), and the remaining quarter did not report the measurement method used. No interventions using Strength Exercise investigated changes in the VSN. Finally, eight interventions (~67%) focused on Mixed Exercise. Of these, one quarter reported Light-to-Moderate Intensity (measured via HR with a range of 50–69% HR_max_), another quarter Vigorous Intensity (measured via PWC with an estimated range of 82–86% HR_max_), while the remaining half did not report any intensity. Among these interventions, the majority (*n* = 6) evaluated Short-Term effects, while two interventions evaluated Long-Term effects.

Of the twelve interventions analyzing the VSN, four studies reported only functional changes, seven reported structural changes, and three documented both.

Functional changes were primarily investigated through resting-state and task-based FMRI. For instance, Lehmann et al. [17] and Tomita et al. [27] reported increased functional connectivity and activation in the posterior parietal cortex, precuneus, and occipito-parietal junction, regions associated with visuospatial attention and integration. These studies highlighted the enhanced engagement of Gray Matter areas within the VSN following Cardiovascular and Mixed Exercise programs. Structural changes were assessed using VBM and DTI. Rehfeld et al. [29] and Duru et al. [32] demonstrated increased Gray and White Matter volumes in VSN-related regions, including the parietal lobes, and improvements in the White Matter microstructure, suggesting enhanced neural efficiency and plasticity in visuospatial processing pathways. Neuroimaging tools included FMRI for functional connectivity and activation mapping, and SMRI, VBM, and DTI for the structural analysis of Gray and White Matter.

The Sensorimotor Network (SMN) is located in the posterior bank of the central sulcus [33]. The involvement of the SMN was reported in thirteen papers [18,20,26,29,30,31,32,39,45,46] consisting of fourteen interventions. Of the fourteen interventions analyzed, four were based on Cardiovascular Exercise (~29%): one quarter employed adopted Light-to-Moderate Intensity, another quarter Vigorous Intensity, and the remaining half did not report the exercise intensity. Most of the interventions focused on Short-Term effects (*n* = 3), while only one did so on Long-Term effects. In one quarter, the intensity was assessed through HR (ranging from 40 to 70%), in another quarter through RPM (60–70 RPM corresponding to 120–170 BPM), and half did not specify the measurement method used. Two interventions (~14%) focused on Strength Exercise and both employed a Vigorous Intensity. One had a load ranging from 80 to 85.5% of 1 RM over a three-week period (Short-Term effect), whereas the other used MVC at 80%, corresponding to 72.2% of 1 RM over an eight-week period (Short-Term effect). Finally, eight interventions (~57%) focused on Mixed Exercise. Among these, one reported Light-to-Moderate Intensity (measured via HR with ranges of 50–60% HR_max_), two used Vigorous Intensity, measured via PWC with an estimated range of 82–86% HR_max_), while five did not report any intensity. Of these interventions, the majority (*n* = 6) evaluated Short-Term effects, while two interventions evaluated Long-Term effects.

Of the fourteen interventions analyzed for the SMN, six papers have shown functional changes; six structural ones; and two both. Regarding the increase in functional changes, papers are primarily concerned with an improvement in the brain areas within the SMN’s connectivity and activation, particularly in Gray Matter and Primary Motor cortex excitability. On the other hand, regarding the increase in structural changes, papers are primarily concerned with an improvement in the brain areas within the VSN’s volume, such as Gray and White Matter and parietal lobes, and an increase in White Matter microstructure changes. The neural investigation tools for enquiring into Primary Motor cortex excitability were TMS and EMG, through motor-evoked potential over M1 and the short-latency intracortical inhibition (SICI) protocol, FMRI, SMRI, or both.

##### Language and Auditory Networks

The Language Network (LN) is considered a functionally distinct group of areas [42,47,48]; connections—enhanced by physical exercise both in number and in action potential—between language-specific and domain-general regions have been found in the left hemisphere, extended to all temporal pole regions (e.g., the poles of the superior, middle, and inferior temporal gyri) [49].

The Auditory Network (AN), which is one of the functional Brain Networks, is part of the cerebral cortex, which is divided into the primary and secondary auditory cortices [33,42,48,50]. Changes regarding the LN and the AN, referring to an integrated action among them, were analyzed in the same fifteen studies [17,18,19,22,23,24,27,28,29,30,32,35,36,37] consisting of seventeen interventions. Out of the seventeen interventions, nine were based on Cardiovascular Exercise (53%). Four interventions used a Light-to-Moderate Intensity, another four used Vigorous Intensity, and one did not report the exercise intensity. Of these, most focused on Short-Term effects (*n* = 8), while only one on Long-Term effects. Six interventions were measured via HR (ranging from 50 to 85% HR_max_), one via RPM (60–70 RPM corresponding to 120–170 BPM), one by VO_2_ (~80% of VO_2max_), and another one did not report the measurement method used. One intervention focused on Strength Exercise and proposed a Vigorous Intensity (70–85% 1 RM) for a period of 52 weeks (Long-Term effect). Finally, seven interventions (~40%) focused on Mixed Exercise. Of these, one reported a Light-to-Moderate Intensity (with a range of 60–69% HR_max_), two Vigorous Intensity (PWC with an estimated range of 82–86% HR_max_), while four did not report any intensity. Of these interventions, the majority (*n* = 6) evaluated Short-Term effects, while one intervention evaluated Long-Term effects.

Of the seventeen interventions analyzing the LN and the AN, four studies reported only functional changes, ten reported structural changes, and three documented both. The hippocampus was found as a key region of change, both functional (Bosch et al. [23]) and structural (Voss et al. [24]). Regarding the increase in functional changes, papers are primarily concerned with an improvement in the brain areas within the LN and the AN’s connectivity and activation, particularly in the hippocampus. On the other hand, regarding the increase in structural changes, papers are primarily concerned with an improvement in the brain areas within the LN and the AN’s volume, such as Gray and White Matter, the Forepart, hippocampus areas, and parietal lobes, an increase in tissue density, vascular plasticity, and White Matter microstructure changes. According to Kleemeyer [36], volume changes in the hippocampus seem to be mediated by underlying changes in volume density. The neural investigation tools were FMRI, SMRI, or both.

##### White Matter, Subcortical Gray Matter, and Corticospinal Tract

White Matter comprises half of the brain and plays a critical and indispensable role in the organization of distributed Brain Networks, allowing for the transfer of information within its fiber bundles [51]. White Matter is influenced by the different types of physical exercise that can induce change in either functional plasticity, enhancing the transfer of information within fiber bundles, or structural plasticity, increasing the volume. White Matter was studied in six papers [17,18,20,27,29,32] consisting of seven interventions. Out of the seven interventions, three were based on Cardiovascular Exercise (43.0%). Two used a Vigorous Intensity, measured via RPM (ranging from 60 to 70 repetitions per minute, corresponding to a range of 120–170 bpm and 59–86% HR_max_), whereas one did not report the exercise intensity or the measurement method used. Each of them focused on Short-Term effects. No intervention focused on Strength Exercise. Finally, four interventions (57.1%) focused on Mixed Exercise. Of these, half reported a Vigorous Intensity (measured via PWC with a range of 82–86% HR_max_), whereas the other half did not report any intensity. Of these interventions, the majority (*n* = 3) evaluated Short-Term effects, while one intervention evaluated Long-Term effects.

Of the seven interventions analyzing White Matter, one study has shown functional changes; four structural ones; and two both. Interestingly, Benedict et al. [18] showed the main effect of the increase in the total volume of White Matter, rather than Gray Matter. The neural investigation tools were FMRI, SMRI, or both.

The exercise-induced plasticity of Gray Matter synapses is increasingly recognized as pivotal for Central Nervous System function and cognition [51] and information processing [37,52,53]. Subcortical Gray Matter has been studied in three papers [27,28,29] consisting of four interventions. All interventions were based on Mixed Exercise. Out of these, half reported a Vigorous Intensity (measured via PWC with a range of 82–86% HR_max_), whereas the other half did not report any intensity. Of these interventions, the majority (*n* = 3) evaluated Short-Term effects, while one intervention evaluated Long-Term effects (25.0%).

Of the four interventions analyzing Subcortical Gray Matter, two papers reported functional changes and two reported structural ones. Regarding the increase in functional changes, papers are primarily concerned with an improvement in the brain areas within Subcortical Gray Matter’s connectivity and activation. Notably, Huang et al. [45] leveraged graph analysis to highlight the role of the thalamus in integrating cortical and subcortical functional patterns. On the other hand, regarding the increase in structural changes, papers are primarily concerned with an improvement in the brain areas within Subcortical Gray Matter’s volume. The neural investigation tools were FMRI, SMRI, or both.

The Corticospinal Tract is the most direct pathway between the cerebral cortex and the spinal cord, with corticospinal axons monosynaptically synapsing onto spinal motor neurons [54]. The Corticospinal Tract plays a major role in the cortical control of spinal cord activity. In particular, it is the principal motor pathway for voluntary movements [55] and plays a fundamental role in the neural control of locomotion [54]. The Corticospinal Tract has been studied only in two papers [29,32] consisting of three interventions. Each of them was based on Mixed Exercise. Out of these, two reported a Vigorous Intensity (measured via PWC with an estimated HR_max_ range of 82–86%), and one did not report any intensity. Each of them evaluated Short-Term effects.

Of the three interventions analyzing the Corticospinal Tract, none reported only functional changes: two reported structural changes and one reported both. Functional changes were described exclusively in Duru et al. [32], which also documented structural alterations. Using resting-state FMRI, the study found increased functional connectivity between the Primary Motor cortex and spinal projections within the Corticospinal Tract, suggesting enhanced corticospinal excitability following a Mixed Exercise intervention. Structural changes were primarily assessed through DTI and VBM. Both Rehfeld et al. [29] and Duru et al. [32] reported increased White Matter integrity and Gray Matter volume in the Corticospinal Tract and adjacent motor-related areas (e.g., precentral gyrus), indicating potential exercise-induced neuroplasticity in motor pathways. Neuroimaging methods included FMRI for functional connectivity and SMRI for structural analysis.

##### Findings on Functional and Structural Changes

In terms of functional-level brain changes, Colcombe et al. [39] reported that six months of Cardiovascular Exercise led to significantly higher levels of task-related activity in older adults. This increase was observed during cognitively challenging tasks performed simultaneously with physical exercise (i.e., dual-task activity) in the DMNs, SNs, ECNs, VSNs, and SMNs. In the studies by Lehmann et al. [17,20] (changes in the DMN, SN, ECN, VSN, SMN, and White Matter), Bosch et al. [23], Voss et al. [24] (changes in the DMN, LN, and AN), Tomita et al. [27] (changes in the DMN, SN, ECN, LN, AN, VSN, SMN, and White and Subcortical Gray Matter), Bar et al. [28] (changes in the DMN, LN, AN, SMN, and Subcortical Gray Matter), Duru et al. [32] (changes in the DMN, SN, ECN, LN, AN, VSN, SMN, White Matter, and Corticospinal Tract), and across one of the two interventions analyzed by Cui et al. [22] (changes in the DMN, SN, ECN, LN, AN, and VSN), it was demonstrated that Cardiovascular, Strength, and Mixed Exercise influence brain function. These influences include modifications in brain activation, connectivity, and excitability in both young (<35 years) and older (≥60 years) individuals. On the other hand, Motes et al. [34] showed a positive reduction in the activation of the prefrontal cortex (PFC) in DMNs, SNs, and ECNs in older people. Liu-Ambrose et al. [38] investigated the effect of Strength Exercise on functional brain changes, particularly focusing on response inhibition processes (i.e., the ability to avoid making automatic, unwanted responses, as previously described) in DMNs, SNs, LNs, and ANs. The authors suggested that twice-weekly Strength Training may positively influence functional plasticity in the brain cortex in a healthy older female (≥60 years) [38]. Goodwill et al. [46] and Hortobágyi et al. [25] found that Strength Exercise may modulate functional changes in the SMN, mainly increasing the excitability of the Primary Motor Cortex. Finally, Huang et al. [45] underlined that Cardiovascular Exercise could produce functional changes such as increased brain connectivity, again in the SMN.

In terms of structural-level brain changes, as mentioned already above, it was demonstrated that Cardiovascular, Strength, and Mixed Exercise may have the kind of effects observed in the hippocampal and parietal lobes and the Forepart and, in general, the Gray and White Matter volume, tissue density (specifically White Matter microstructure changes), and vascular plasticity with cerebral perfusion. In the study conducted by Cui et al. [22] across two interventions (changes in the DMN, SN, ECN, LN, AN, and VSN), as well as those by Rehfeld et al. [29] (changes in the DMN, SN, ECN, LN, AN, VSN, SMN, White and Subcortical Gray Matter, and Corticospinal Tract), Erickson et al. [37], Voss et al. [24], Kleemeyer et al. [36], Maass et al. [19], Thomas et al. [35] (changes in the DMN, LN, and AN), Benedict et al. [18] (changes in the DMN, SN, ECN, LN, AN, VSN, SMN, and White Matter), Ruscheweyh et al. [30] (changes in the DMN, SN, ECN, VSN, and SMN), Duru et al. [32] (changes in the DMN, SN, ECN, LN, AN, VSN, SMN, White Matter, and Corticospinal Tract), Sampaio-Baptista et al. [31] (changes in the DMN, SN, ECN, LN, AN, VSN, and SMN), and Fukuo et al. [26] (changes in the VSN and SMN), an increased brain volume was observed. These increases mitigate physiological age-related losses in the hippocampal and parietal lobes and the Forepart, and, in general, in Gray and White Matter. These changes occurred in both young adults (<35 years) and older people (≥60 years). Moreover, Kleemeyer et al. [36] observed changes in tissue density, while Lehmann et al. [17,20] observed White Matter microstructure changes that influenced each studied network. These changes were independent of age. With respect to vascular plasticity, Maass et al. [19] confirmed that Cardiovascular Exercise, with a Vigorous Intensity, enhances cerebral perfusion in older people (≥60 years), even when conducted over a Short-Term duration. Finally, only in both Rehfeld et al.’s [29] training interventions and Duru et al. [32], structural changes in the Corticospinal Tract motor neurons, in addition to each of the analyzed networks, as previously described, were found.

**Figure 1:**
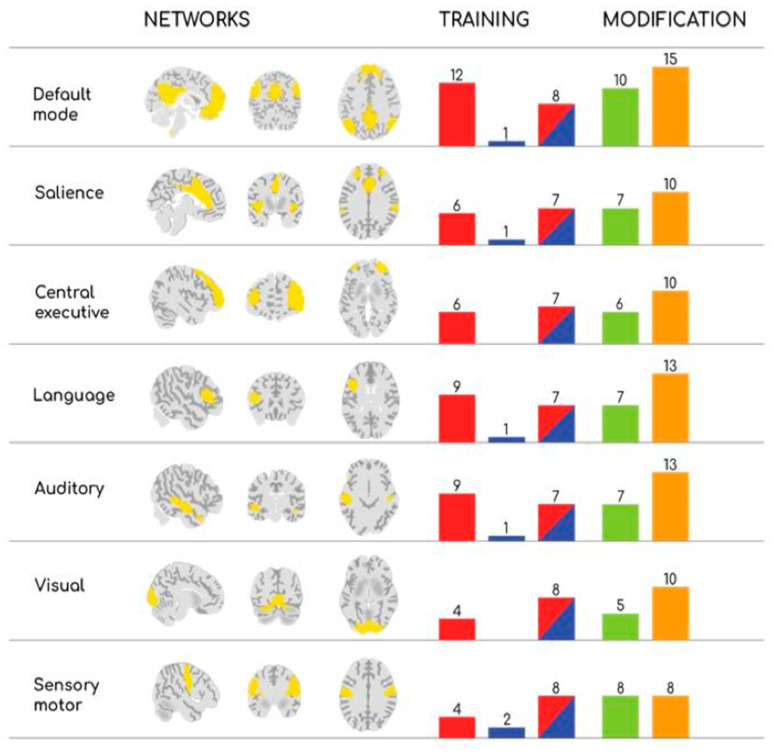
Graphical representation of structural and functional modifications in the seven Brain Networks, as reported in the reviewed studies, in response to three types of exercise training. For each network, the bar graphs indicate the number of studies reporting changes following Cardiovascular (red), Strength (blue), and Mixed Training (red/blue). Bars are further subdivided to reflect functional (green) and structural (orange) changes. Bar heights are proportional to the frequency of reported findings across the included studies. Please note that networks were depicted in neurological standard (left is left) and obtained using the tool Neurosynth (https://neurosynth.org) (accessed on 30 June 2025) which is based on a public platform created for large-scale, automated synthesis of Functional Magnetic Resonance Imaging (FMRI) data. For further details about the platform, see [56].

Figure 1 provides an original graphical description of both structural and functional changes in the seven analyzed Brain Networks as a consequence of the three types of training. Figure 1 is thought to graphically offer the information provided in Table 1, quantifying the occurrences among the reviewed papers. Each of the seven networks is plotted in the three standard views (left is left).) (in Figure 1’s caption more details with respect to the quoted website are reported).

## 4. Discussion

The aim of this article was to provide an overview of research on the effects of physical exercise on brain neuroplasticity using a Brain Network framework. To do this, we identified 25 interventions addressing different exercise types (i.e., Cardiovascular, Strength, and Mixed Exercises) in healthy populations aged 18 to 80 years. Accordingly, the discussion of this narrative review is structured around these three core exercise modalities and then converges on a comparative analysis of how intensity, duration, and population characteristics shape both structural and functional Brain Network adaptations.

### 4.1. Cardiovascular Exercise and Brain: General Overview

Cardiovascular Exercise has been shown to induce changes in brain structure and function, including cognitive benefits [57]. At a structural level, several studies demonstrate that prolonged Cardiovascular Exercise increases the volume of the hippocampus, particularly the right side, and prevents atrophy of the medial temporal lobe and anterior cingulate cortex [15,57,58]. Other studies show that Cardiovascular Exercise combined with task-specific activity can induce neuroplasticity and neuronal proliferation in the prefrontal and parietal areas, which are responsible for the automatic control of motor movements, thus facilitating the brain’s ability to modify its structure and functions [59]. Accordingly, Cardiovascular Exercise can improve the automaticity (i.e., execution of movements without thinking about them) acting on the basal ganglia and their connections, inducing the improvement of executive functions, altered because of dopamine depletion, and generate functional changes in motor learning-related brain structures [59,60]. Moreover, Cardiovascular Exercise promotes the proliferation of neurons and greater activity in the cortical and subcortical areas. For example, Vigorous-Intensity exercise leads to an increase in the excitability of the motor cortical cortex, resulting in improvements in walking parameters (e.g., muscle weakness, reduced aerobic capacity, gait impairment and speed, and balance disorders and falls) and increased activity in the lateral premotor cortex, stimulating the use of alternative Brain Networks [60]. Cardiovascular Exercise could also produce changes in blood flow and vascularity, leading to a better supply of oxygen and nutrition to the brain and increased brain-derived neurotrophic factor levels [57]. Again, Cardiovascular Exercise practiced regularly may increase brain connectivity (i.e., in the Brain Networks analyzed in this paper), resulting in a lower cognitive decline and, consequently, in a general improvement in cognitive functions [57,59]. Finally, it reduces the levels of inflammatory markers, providing an anti-inflammatory effect [15].

### 4.2. Strength Exercise and Brain: General Overview

It has been demonstrated that the increase in muscle function, induced by Strength Exercise, is often accompanied by neuroplastic adaptations in the CNS [25,61,62]. As said before, maintaining the functional plasticity of the cortex is essential for healthy aging, and Resistance Training, like Aerobic Exercise, has been shown to offer similar benefits for the functional plasticity of the brain in older adults [38]. For example, in a group of older females, 12 months of twice-weekly Resistance Training was shown to produce changes in functional plasticity in two cortical regions, the anterior part of the left middle temporal gyrus and the left anterior insula extending into the lateral orbital frontal cortex, associated with response inhibition processes (i.e., the ability to avoid automatic, unwanted responses) [38]. These changes co-occurred with improved flanker task performance (i.e., a task that engages both selective attention and conflict resolution), enhanced by Resistance Training in two ways: first, through an increased engagement of response inhibition processes when needed, and, on the other hand, a decreased tendency to prepare response inhibition as a default state. However, these Strength Exercise effects were observed only when practiced at least twice a week [38]. Finally, at a structural level, Resistance Training increases the hippocampus volume and reduces the levels of inflammatory markers [15].

### 4.3. Mixed Exercise and Brain: General Overview

Different activities were proposed in this type of intervention, including Aerobic and Resistance Training. Evidence shows that this Mixed intervention induces a possible neuroprotective effect [60].

For example, Tai Chi has been seen to increase the volume of Gray Matter [14,15]. Again, the combination of multiple exercise types within a single training session (e.g., flexibility, strength, balance, coordination, and aerobic exercise) has been associated with variations in structural plasticity, such as an increase in the Gray Matter volume, correlated with improvements in physical function [60]. Interestingly, other Mixed interventions such as dancing activity may increase the Gray as well as White Matter volume, suggesting that this type of exercise may have an additional positive influence in promoting such changes [13].

## 5. Conclusions

Overall, the findings suggest that the type, intensity, and duration of physical exercise could lead to neural modification over time. Specifically, exercise intervention contributes to both the brain’s structure (e.g., increase in brain volume, particularly in the Gray and White Matter, hippocampus, and frontal and parietal lobes; increase in tissue density, such as White Matter microstructure; and vascular plasticity, increasing cerebral blood flow) and functional changes (e.g., decrease in PFC activation; increased brain connectivity, excitability of Primary Motor Cortex, and activation, particularly in the hippocampus and Gray Matter). These structural and functional changes were observed in key networks such as the Default Mode Network (DMN), Salience Network (SN), Central Executive Network (ECN), Visuospatial Network (VSN), Sensorimotor Network (SMN), and Language and Auditory Networks (LN and AN).

Nevertheless, such improvements were observed both at the functional and structural levels; the reviewed articles reported non-uniform results, suggesting that several factors may influence these outcomes. Differences in exercise modalities along with varying levels of exercise intensity and duration and the age or health status of participants could contribute to these diverse effects. Indeed, out of twenty-five interventions, nine (36%) reported functional changes [23,25,27,28,34,38,39,45,46], twelve (48%) documented structural changes [17,18,19,22,26,29,30,31,35,36], whereas four (16%) noted both functional and structural modifications [20,22,24,32]. As a general outcome, functional changes from exercise were found in the percentage range of 33–56% (with the average value of 39%), and with respect to structural changes, throughout the observed networks (see Figure 1).

Taken together, these results underline the complexity of understanding how different exercise regimens impact Brain Networks across different populations. When compared with previous works, the findings collected herein provide a more general description of the topic. For example, in the review of Li et al. [63], it was shown that Aerobic Exercise may increase left/right anterior hippocampal volumes compared to the no-exercise interventions, with consequential functional activation or connectivity; such a modification was observed only within the DMN [63]. Similarly, dancing, which is a form of Mixed Exercise, was the single focus of another review, where an improved connectivity between the hemispheres was found [64]. Finally, in the work of Bray et al. [65], only functional improvements were observed in Brain Network connectivity, and such modifications were more evident in healthy and cognitively impaired older adults.

From the perspective of exercise, variability was observed in terms of the type of exercise intervention, intensity, duration, and frequency (minutes per week, days per week, although this fourth factor was not analyzed in this context). The most used training protocol among the twenty-five interventions analyzed was based on Cardiovascular Exercise [17,18,19,20,22,23,24,34,35,36,37,39,45] (52%), followed by Mixed [22,26,27,28,29,30,31,32] (36%), and Strength Exercise [25,38,46] (12%). Regarding intensity, excluding the seven interventions (28%) where intensity was not reported [18,26,27,28,31,32,45], and considering studies conducted by Cui et al. [22] across two interventions, as well as those by Rehfeld et al. [29], Vigorous Intensity was the most prevalent (ten interventions, ~56%) [17,19,20,25,29,35,36,38,46], compared to the Light-to-Moderate used in eight interventions (~45%) [22,23,34,37,39]. Finally, in terms of intervention duration, excluding the one intervention [18] where duration was not reported, such a factor ranged from 2 weeks to 15 years, with 2 months being the most common length.

In conclusion, the evidence presented herein underscores the beneficial effects of exercise on the structural and functional integrity of the brain, highlighting its importance as a non-pharmacological intervention for improving cognition. Our analysis does not suggest a specific type of physical exercise, but it aimed to underline that the regular practice of physical exercise, understood as any routine that promotes physical well-being, has an observable and measurable impact on Brain Networks.

## 6. Limitation and Future Directions

It is necessary to point out that our approach has some limitations. The large variability in exercise parameters as well as in target population must be carefully considered as issues when interpreting these findings. While a narrative review offers flexibility and facilitates the identification of emerging trends, it presents limitations including the potential for selection bias, the absence of formal quality assessment, and the lack of quantitative synthesis. Consequently, although we focused on major biomedical databases and applied criteria to categorize studies according to exercise type, intensity, and duration, we did not adopt a systematic screening protocol (e.g., randomized controlled trials, longitudinal prospective studies, or observational and cross-sectional designs). This lack of stratification may affect the interpretability and strength of the conclusions, as studies with different methodological rigor can yield findings of varying reliability. We also note that the study selection was conducted by a single reviewer, with a second author consulted in case of uncertainty, an approach that, while structured, may not fully eliminate the risk of subjective bias. Consequently, the synthesis presented should be interpreted with caution, considering this methodological heterogeneity.

From a practical point of view, our analysis highlights the need for longitudinal studies exploring the Long-Term effects of different exercise modalities on brain plasticity, as well as in middle-aged populations, to better understand the specific effects of exercise on Brain Networks and functions. Again, future studies should further investigate how different types of motor skills—particularly open- vs. closed-skill activities—differentially affect the structure and function of Brain Networks, especially those involving the VSN and SMN. This line of research may help to clarify the specific neuroplastic mechanisms associated with various exercise modalities, as suggested by recent findings [66,67]. Finally, we suggest that future research efforts include a more systematic appraisal of study design and quality, particularly considering the growing interest in evidence-based interventions for brain health, and adopt different training intensities and durations according to the ACSM indications, allowing for a higher rate of feasibility in the assessment of the causality relationship between exercise and Brain Network modifications.

## Figures and Tables

**Table 1:** Summary of articles according to study characteristics and network domain outcomes. Data are presented considering the type of exercise (i.e., Cardiovascular, Strength, and Mixed Exercises), intensity (i.e., Light-to-Moderate or Vigorous Intensity), and duration (Short-Term and Long-Term Effects).

N.	AUTHORS, YEARS	PARTICIPANT (NUMBER, AGE, GENDER)	EXERCISE CHARACTERISTICS				OBSERVED CHANGES				BRAIN NETWORKS							SUBCORTICAL GRAY MATTER	WHITE MATTER	CORTICO SPINAL TRACT
			TYPE	INTENSITY	DURATION	FREQUENCY (N/WK)	MODIFICATIONS	FUNCTIONAL	STRUCTURAL	TYPE	DMN	SN	ECN	LN	AN	VSN	SMN			
Cardiovascular Exercise																				
Light-to-Moderate Intensity—Short-Term Effect																				
1	Bosch [23] 2021	*n*= 18 Age = 23 yrs F = 0%	Cycling	MI: 65% HRmax HI: 50–75% HRmax	MI: 30 min HI: 15 min	12 wks 1 session	↑ Hippocampal activation	X		FMRI	X			X	X					
2	Colcombe [39] 2004	*n* = 29 Age = 66 yrs F = 37.9%	Walking	40–70% HRmax	40–45 min	3/wk	↑ Activation	X		FMRI	X	X	X			X	X			
3	Cui [22] 2019	*n* = 12 Age = 22 yrs F = 83.3%	Walking	60–69% HRmax	60 min	8 wks–3/wk	↑ Gray Matter volume		X	SMRI	X	X	X	X	X	X				
4	Erickson [37] 2011	*n* = 120 Age = 68 yrs F = 66.6%	Walking	50–75% HRmax	40 min	3/wk	↑ Forepart volume		X	SMRI	X			X	X					
5	Motes [34] 2018	*n* = 41 Age = 63 yrs F = 71.4%	Walking Cycling	50–75% HRmax	60 min	12 wks–3/wk	↓ PFC activation	X		FMRI	X	X	X							
**Light-to-Moderate Intensity—Long-Term Effect**																				
6	Voss [24] 2013	*n* = 65 Age = 66 yrs F = 72%	Walking	50–75% HRmax	40 min	52 wks–3/wk	↑ Connectivity ↑ Hippocampal volume	X	X	FMRI SMRI	X			X	X					
**Vigorous Intensity—Short-Term Effect**																				
7	Kleemeyer [36] 2016	*n* = 52 Age = 66 yrs F = 61.5%	Cycling	HI:80% VO_2max_ LI: 60–90 RPM	25 to 55 min	3 wks 2/wk 21 wks 3/wk	↑ Microstructural (tissue density) ↑ Volume		X	SMRI	X			X	X					
8	Lehmann [17] 2020	*n* = 31 Age = 23 yrs F = 60%	Cycling	60–70 RPM 120–170 BPM (59–86% HRmax) *	Week 1: 19 min Week 2: 21 min	2 wks 7 sessions	↑ Cerebral blood flow ↑ White Matter microstructure changes		X	FMRI SMRI	X	X	X	X	X				X	
9	Lehmann [20] 2022	*n* = 31 Age = 23 yrs F = 60%	Cycling	60–70 RPM 120–170 BPM (59–86% HRmax) *	Week 1: 19 min Week 2: 21 min	2 wks 7 sessions	↑ White Matter microstructure changes ↑ Gray Matter activation	X	X	FMRI SMRI	X	X	X			X	X		X	
10	Maass [19] 2015	*n* = 40 Age = 69 yrs F = 55%	Walking Running	65–80% HRmax	30 min	18 wks 3/wk	↑ Blood flow (vascular plasticity) ↑ Volume		X	SMRI	X			X	X					
11	Thomas [35] 2016	*n* = 62 Age = 34 yrs F = 56.4%	Cycling	55–85% HRmax	30 min	6 wks 5/wk	↑ Anterior hippocampus volume		X	SMRI	X			X	X					
**No Reported Intensity—Short-Term Effect**																				
12	Benedict [18] 2013	*n* = 331 Age = 75 yrs F = 49.5%	Running Swimming Cycling Walking	n.r.	30 min minimum	n.r.	↑ White Matter ↑ Gray Matter Parietal lobes volume		X	SMRI	X	X	X	X	X	X	X		X	
**No Reported Intensity—Long-Term Effect**																				
13	Huang [45] 2017	*n* = 30 Age = 18–29 yrs F = 53.3%	Swimming	n.r.	n.r.	15 yrs minimum	↑ Connectivity	X		FMRI							X			
**Strength Exercise**																				
**Vigorous Intensity—Short-Term Effect**																				
14	Goodwill [46] 2012	*n* = 14 Age = 21 yrs F = 50%	Single right leg squats	4X6 80–85% 1 RM	n.r.	3 wks 3/wk	↑ Excitability Primary Motor cortex	X		TMS + EMG							X			
15	Hortobágyi [25] 2011	*n* = 20 Age = 31 yrs F = 40%	Isometric right-hand first dorsal interosseous muscle contractions	80% MVC	15–20 min 5 blocks of 10	8 wks 20 sessions	↑ Excitability Primary Motor cortex	X		TMS + EMG							X			
**Vigorous Intensity—Long-Term Effect**																				
16	Liu-Ambrose [38] 2012	*n* = 52 Age = 69 yrs F = 100%	Mini squats, mini lunges, lunge walk with both a Keiser Pressurized Air System and free weights	2X6-8 7-RM method (70–85% 1 RM) *1	n.r	52 wks 2/wk	↑ Functional changes	X		FMRI	X	X		X	X					
**Mixed Exercise**																				
**Light-to-Moderate Intensity—Short-Term Effect**																				
17	Cui [22] 2019	*n* = 12 Age = 22 yrs F = 83.3%	Tai Chi	60–69% HRmax	60 min	8 wks 3/wk	↑ Connectivity ↑ Gray Matter volume	X	X	FMRI SMRI	X	X	X	X	X	X				
18	Ruscheweyh [30] 2011	*n*= 62 Age = 60 yrs F = 69.3%	Nordic walking/stretching exercises	Nordic walking 50–60% HRmax Stretching exercises 30–40% maximal exertion	50 min	24 wks 3/wk up to 5/wk	↑ Gray Matter volume		X	SMRI	X	X	X			X	X			
**Vigorous Intensity—Short-Term Effect**																				
19	Rehfeld [29] 2018	*n* = 20 Age = 68 yrs F = 60%	Dancing	PWC130 (82–85.5% HRmax) *	90 min	24 wks 2/wk	↑ White and Gray Matter volume		X	SMRI	X	X	X	X	X	X	X	X	X	X
20	Rehfeld [29] 2018	*n* = 18 Age = 69 yrs F = 44%	Cycling/strength endurance/flexibility	PWC130 (83–86% HRmax) *	90 min	24 wks 2/wk	↑ White and Gray Matter volume		X	SMRI	X	X	X	X	X	X	X	X	X	X
**No Reported Intensity—Short-Term Effect**																				
21	Bar [28] 2016	*n* = 5 Age = 28 yrs F = 0%	Dancing	n.r.	n.r.	34 wks 36 rehearsal sessions	↑ Activation	X		FMRI	X			X	X		X	X		
22	Duru [32] 2018	*n* = 13 Age = 22 yrs F = 46%	Karate	n.r.	n.r.	10 yrs minimum 15 h/wk	↑ White and Gray Matter volume ↑ Connectivity	X	X	FMRI SMRI	X	X	X	X	X	X	X		X	X
23	Sampaio-Baptista [31] 2014	*n* = 40 Age = 24 yrs F = 55%	Juggling	n.r.	15 or 30 min	6 wks 5/wk	↑ Volume		X	SMRI	X	X	X	X	X	X	X			
**No Reported Intensity—Long-Term Effect**																				
24	Fukuo [26] 2020	*n*= 10 Age = 20 yrs F = 0%	Artistic gymnastic exercises	n.r.	n.r.	13.6 ± 2.2 years of training	↑ Gray Matter volume		X	SMRI						X	X			
25	Tomita [27] 2021	*n* = 10 Age = 20 yrs F = 0%	Artistic gymnastic exercises	n.r.	n.r.	13.6 ± 2.2 years of training	↑ Connectivity	X		FMRI	X	X	X	X	X	X	X	X	X	

Notes: DMN, Default Mode Network; SN, Salience Network; ECN, Executive Control Network; LN, Language Network; AN, Auditory Network; VSN, Visuospatial Network; SMN, Sensorimotor Network; SMRI, Structural Magnetic Resonance Imaging; FMRI, Functional Magnetic Resonance Imaging; TMS, Transcranial Magnetic Stimulation; EMG, Electromyography; HRmax, Maximum Heart Rate; EG, Experimental Group; CG, Control Group; VO_2_max, Maximal Oxygen Uptake; RPM, Repetitions Per Minute; RM, Repetition Maximum; MVC, Maximum Voluntary Contraction; BPM, Beats Per Minute; PFC, Prefrontal Cortex; PWC, Physical Working Capacity; LIPA, Light Intensity Physical Activity; MVPA, Moderate to Vigorous Physical Activity; EEG, Electroencephalogram; n.r., Not Reported; and *: HRmax was calculated indirectly when not available by age and BPM through HRmax theoretical formula. *1: 6–8 repetitions of 1 RM are in the hypertrophic strength range (70–85% 1 RM) ↑ indicates an increase (or improvement); ↓ indicates a decrease (or reduction); *n*, number of subjects.

## Data Availability

No new data were created or analyzed in this study. Data sharing is not applicable to this article.
